# Bilateral cornu ammonis 3–dominant mechanism of visual working memory impairment in right temporal lobe epilepsy and hippocampal sclerosis

**DOI:** 10.1093/braincomms/fcag284

**Published:** 2026-07-20

**Authors:** Qiao Wang, Xiaotian Qi, Chao Liu, Linan Hou, Cheng Wang, Yongqi Cheng, Zhengye Yu, Chao Han, Ximu He

**Affiliations:** Department of Pathology, Wuhu Hospital, East China Normal University (The Second People’s Hospital of Wuhu), Wuhu 241000, China; Department of Pathology, Wuhu Hospital, East China Normal University (The Second People’s Hospital of Wuhu), Wuhu 241000, China; Department of Neurology, Deyu Medical Maanshan General Hospital, Ma’anshan 243000 Anhui, China; Department of Neurology, Lixin County People’s Hospital, Bozhou 236702, China; Department of Neurology, Wuhu Hospital, East China Normal University, Wuhu 241001, China; Department of Neurology, Wuhu Hospital, East China Normal University, Wuhu 241001, China; Department of Neurology, Wuhu Hospital, East China Normal University, Wuhu 241001, China; Department of Neurology, The First Affiliated Hospital of USTC, Division of Life Sciences and Medicine, University of Science and Technology of China, Hefei 230001, China; The School of Psychology and Cognitive Science, East China Normal University, Shanghai 200062, China

**Keywords:** CA3, cognitive dysfunction, epilepsy, hippocampal sclerosis

## Abstract

Hippocampal sclerosis in temporal lobe epilepsy is linked to cognitive impairment. This study investigates its exacerbating role in visual working memory deficits in right temporal lobe epilepsy and the involvement of contralateral hippocampal subregions. We enrolled 103 patients with right temporal lobe epilepsy (57 with right hippocampal sclerosis and 46 without hippocampal sclerosis) and 36 healthy controls who underwent high-resolution MRI and neuropsychological assessment. Patients with hippocampal sclerosis showed more severe visual working memory impairment, widespread grey matter atrophy and significant volume loss in bilateral cornu ammonis 3 and right dentate gyrus. Contralateral cornu ammonis 3 atrophy correlated with poorer visual working memory performance. Hippocampal sclerosis in right temporal lobe epilepsy is linked to severe visual working memory deficits and extensive bilateral hippocampal atrophy. These findings establish hippocampal sclerosis as a key biomarker and underscore the role of bilateral hippocampal network disruption, with contralateral cornu ammonis 3 integrity influencing cognitive outcomes.

## Teaser

Wang *et al*. report that in right temporal lobe epilepsy, the presence of hippocampal sclerosis is associated with more severe visual working memory deficits. This impairment correlates with atrophy not only in the ipsilateral hippocampus but also critically in the contralateral CA3 subregion, highlighting a bilateral network disruption impacting cognition.

## Graphical Abstract

**Graphical Abstract:**
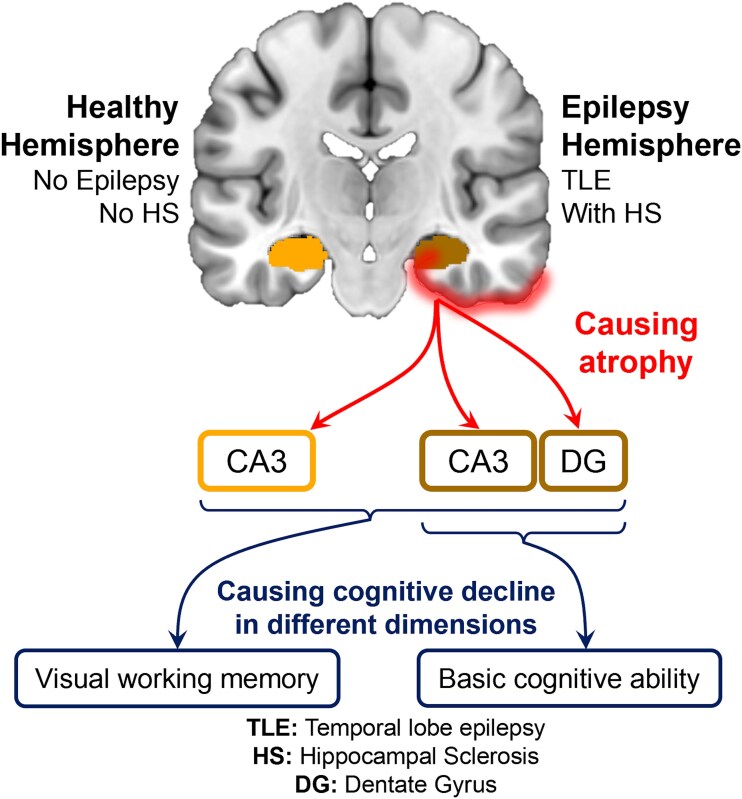

## Introduction

Temporal lobe epilepsy (TLE) is the most common drug-resistant epilepsy in adults, primarily characterized by pathological alterations in mesial temporal lobe structures, most frequently involving the hippocampus.^1,2^ A key neuropathological hallmark of TLE is hippocampal sclerosis (HS).^3^ Simultaneously, the degree of hippocampal atrophy is known to correlate with the severity of cognitive impairment.^4^ Consequently, brain MRI is routinely employed for the non-invasive detection of HS in the diagnostic evaluation of TLE. Studies of drug-resistant TLE cohorts have consistently demonstrated significantly reduced volumes in the ipsilateral hippocampus and its subregions compared to both the contralateral side and healthy controls.^5^ The conceptualization of epilepsy as a network disorder helps explain how focal temporal lobe seizures can disrupt both local and remote brain structures.^6,7^ While ipsilateral hippocampal damage is well-documented, the impact on the contralateral hippocampus and the broader implications of these bilateral network effects remain less clear and require further investigation.

TLE and HS are strongly associated with memory impairments, believed to stem from structural damage that disrupts the hippocampus's critical role in memory processing.^8,9^ However, there is significant heterogeneity present, as not all TLE patients exhibit memory deficits.^10^ This variability may be critically linked to the differential vulnerability of hippocampal subregions. Structurally and functionally, the hippocampus is not uniform but comprises distinct subregions [e.g. cornu ammonis sectors CA1–4 and the dentate gyrus (DG)],^11^ which contribute variably to memory processes.^12,13^ The predominant information flow through these circuits follows the canonical tri-synaptic pathway: from the medial entorhinal cortex (mEC) to the DG, then to CA3 and finally to CA1,^14^ which is involved in the initial encoding and retention periods of visual working memory (VWM).^15^ Therefore, the precise pattern of hippocampal subregion atrophy in HS, rather than overall hippocampal volume, may determine the profile and severity of memory impairment.

Meanwhile, VWM deficits are a prominent and clinically significant feature of TLE.^16,17^ As a core cognitive system for the temporary maintenance and manipulation of information, VWM is fundamental to higher-order cognition.^18,19^ Notably, the neural substrates supporting VWM, particularly for non-verbal/visual information, show a degree of hemispheric lateralization, with right hemispheric regions playing a dominant role.^20-22^ However, the VWM decline associated with hippocampal subregion-level atrophy remains unclear in the context of right TLE (rTLE) with HS. The structural bases of VWM deficits in TLE, particularly in relation to subregion-specific pathology in a lateralized cohort, are not well characterized. To more directly and sensitively investigate the impact of hippocampal subregion atrophy in HS on this cognitive domain while controlling for material-specific lateralization confounds, we focused on patients with rTLE. This approach allows a clearer examination of how pathology in the hippocampal subregions predominantly engaged by the chosen cognitive task relates to VWM behavioural performance.

Therefore, this study aimed to: (i) characterize the patterns of grey matter (GM) and bilateral hippocampal (especially subregional) atrophy in patients with rTLE and HS using high-resolution MRI; (ii) examine whether the presence of HS exacerbates VWM impairment on a delayed matching-to-sample (DMS) task^23^; and (iii) analyse the correlations between subregion-specific atrophy patterns and behavioural performance, in order to elucidate how distinct hippocampal subregion pathologies contribute to VWM deficits in rTLE.

## Materials and methods

### Participants

A total of 103 patients diagnosed with rTLE were recruited from Wuhu Hospital affiliated to East China Normal University. The inclusion criteria were as follows: (i) diagnosis of rTLE according to the 2017 International League Against Epilepsy classification criteria^24^; (ii) confirmation of rTLE through a comprehensive evaluation encompassing seizure semiology, EEG and MRI; (iii) right-handedness; and (iv) provision of written informed consent and willingness to complete all study procedures. HS was identified based on established MRI features, specifically findings on T2-weighted or FLAIR sequences. Exclusion criteria included: (i) concurrent neurological or psychiatric disorders, or the presence of secondary cognitive impairment; (ii) severe systemic comorbidities affecting the cardiovascular, cerebral, renal, or hepatic systems; and (iii) inability to complete the neuropsychological assessments or to comply with the experimental protocol. Additionally, 36 age- and sex-matched healthy controls (CON) were recruited from individuals undergoing routine health examinations at Wuhu Hospital affiliated to East China Normal University. The study protocol was approved by the institutional review board of Wuhu Hospital affiliated to East China Normal University. This study was conducted in accordance with the principles of the Declaration of Helsinki. All participants provided written informed consent prior to enrolment.

Participants were divided into three groups: patients with rTLE and right HS (RR group, *n* = 57), patients with rTLE without HS (RN group, *n* = 46) and CON group (*n* = 36). The RR group consisted of 38 males and 19 females, with a mean age of 37.18 ± 12.93 years, a median disease duration of 8.95 years [interquartile range (IQR) = 5.61] and a mean education of 13.53 ± 2.03 years. The RN group included 27 males and 19 females, with a mean age of 29.37 ± 9.25 years, a median disease duration of 3.98 years (IQR = 2.09) and a mean education of 15.02 ± 1.69 years. The CON group comprised 16 males and 20 females, with a mean age of 31.64 ± 8.85 years and a mean education of 14.56 ± 2.1 years. Detailed demographic and clinical characteristics are presented in Table 1.

**Table 1:** Baseline characteristics of the study participants

Items	CON (*n* = 36)	RN (*n* = 46)	RR (*n* = 57)	CON versus RN (*P*-value)	CON versus RR (*P*-value)	RN versus RR (*P*-value)
**Demographics**						
Age [year, *M* (IQR)]	27.5 (11)	26 (11)	33 (21)	0.577^a^	0.217^a^	0.002^a^
Male/female (*n*)	16/20	27/19	38/19	0.200^b^	0.034^b^	0.405^b^
Education [year, *M* (IQR)]	15 (3)	16 (1)	12 (4)	0.163^a^	0.678^a^	0.002^a^
**Clinical features**						
Disease duration [year, *M* (IQR)]	—	3 (1)	7 (10)	—	—	*<*0.001^c^

^a^Kruskal–Wallis test with Bonferroni-adjusted *post hoc* comparisons.

^b^Chi-squared test.

^c^Mann–Whitney U-test.

### Neuropsychological assessment

All participants completed a comprehensive neuropsychological assessment administered by an experienced clinical psychologist blinded to all clinical and imaging data. The assessment included evaluation of basic cognitive function using the Montreal Cognitive Assessment (MoCA) and the Mini-Mental State Examination (MMSE). VWM was measured via a DMS task, yielding two outcome measures: reaction time (VWM_RT, the average time taken to respond) and accuracy (VWM_ACC, the percentage of correct responses).^23^

### VWM task

**Figure 1:**
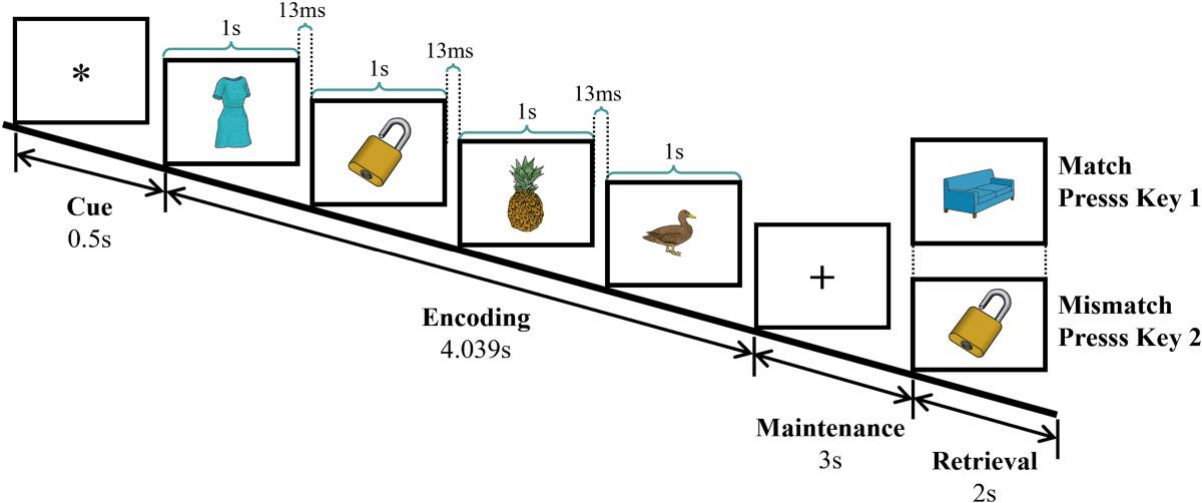
**Visual working memory paradigm.** Each trial was initiated by a 0.5-s central fixation point (‘*’), followed by the encoding phase in which four images were presented sequentially (1-s duration each; 13-ms interstimulus interval). Subsequently, a cue (‘+’) marked the start of the 3-s maintenance phase. In the final retrieval stage, a probe image was displayed, and participants had 2 s to indicate whether it matched any image from the previously encoded memory set. The example object stimuli displayed in the paradigm are reproduced from Rossion and Pourtois (2004).

Participants completed a visual DMS task to assess VWM. Each trial began with a central fixation cross (‘+’) for 0.5 s, followed by sequential encoding of four images from the Snodgrass and Vanderwart set (1 s each, 13-ms interval).^23^ After a 3-s maintenance period, a probe image appeared, and participants had 2 s to indicate whether it matched any encoded image. The task comprised 60 trials across six blocks. Performance was measured by VWM_RT and VWM_ACC. A schematic of the task is shown in Fig. 1.

### MRI protocol

MRI data were acquired using a 3-Tesla Siemens TrioTim scanner equipped with a Head Matrix head coil and an eight-channel brain phased-array coil. T1-weighted (T1w) images were obtained in the sagittal plane with a three-dimensional magnetization-prepared rapid gradient-echo (3D-MPRAGE) sequence. The imaging parameters were as follows: voxel size = 1 mm × 1 mm × 1 mm, repetition time = 1600 ms, echo time = 2.52 ms, inversion time = 900 ms, flip angle = 9°, matrix = 256 × 240 × 176, bandwidth = 170 Hz/pixel. High-resolution T2-weighted hippocampal (T2-Hipp) images were acquired in the coronal orientation using a two-dimensional turbo spin echo sequence with the following parameters: voxel size = 0.28 mm × 3.3 mm × 0.28 mm, repetition time = 3600 ms, echo time = 94 ms, flip angle = 120°, matrix = 320 × 320, number of slices = 20, bandwidth = 220 Hz/pixel.

### MRI data preprocessing

T1w structural images were preprocessed using Statistical Parametric Mapping (SPM12).^25^ The pipeline included bias field correction; segmentation into GM, white matter and cerebrospinal fluid; and spatial normalization of the resulting GM probability maps to the Montreal Neurological Institute (MNI) standard space. The normalized GM images were then modulated to preserve the total amount of GM tissue and resampled to an isotropic resolution of 1.5 mm for subsequent voxel-based morphometry (VBM) analysis. For the automated segmentation of hippocampal subregions, T2-Hipp images were processed alongside the T1w images using HippUnfold v1.5.2.^26^ All hippocampal subfield segmentations generated by HippUnfold were visually inspected by a trained rater to ensure anatomical accuracy, particularly for the small CA3 subregion. This pipeline provided the estimated GM volume (GMV) for each predefined subregion within the bilateral hippocampus. These subregional volume estimates served as the basis for the region-of-interest (ROI) analysis and subsequent correlation analyses.

### Statistical analysis

#### Demographic and clinical characteristics

Statistical analyses of demographic and clinical data were performed using SPSS version 20.0 (SPSS Inc., Chicago, IL, USA), with a significance level set at *P* <0.05 (two-tailed). Normality of continuous variables was assessed using the Shapiro–Wilk test. Continuous variables are presented as mean ± standard deviation or median (IQR), as appropriate, while categorical variables are presented as counts and percentages. Between-group differences in demographic and clinical characteristics were evaluated using appropriate statistical tests. The Kruskal–Wallis test was employed to compare continuous variables (e.g. age and years of education) across the three groups (CON, RN and RR), followed by Bonferroni-corrected *post hoc* pairwise comparisons where applicable. Disease duration between the two patient groups (RN and RR) was compared using the Mann–Whitney U-test. The chi-squared test was used to examine differences in sex distribution among the groups.

#### Neuroimaging data analysis

Whole-brain, voxel-wise group comparisons of GMV were conducted using a one-way analysis of covariance (ANCOVA) model in SPM12. A group-level GM mask was created by thresholding (*P* = 0.3) and binarizing the default GM tissue probability map provided in SPM12. Statistical maps were thresholded at a cluster-level significance of *P* <0.05, corrected for multiple comparisons using the false discovery rate (FDR), with an underlying cluster-forming threshold of 60 contiguous voxels.

For both the whole-brain VBM and the ROI analyses of hippocampal subregions, general linear models were employed. These models included the diagnostic group as the factor of interest and were adjusted for the following covariates: age, sex, years of education and total intracranial volume (TIV). The parameter estimates from these models were used to derive adjusted volumetric values for group comparisons and for the subsequent partial correlation analysis with cognitive scores.

## Results

### Widespread and region-specific GM atrophy after covariate adjustment

**Figure 2:**
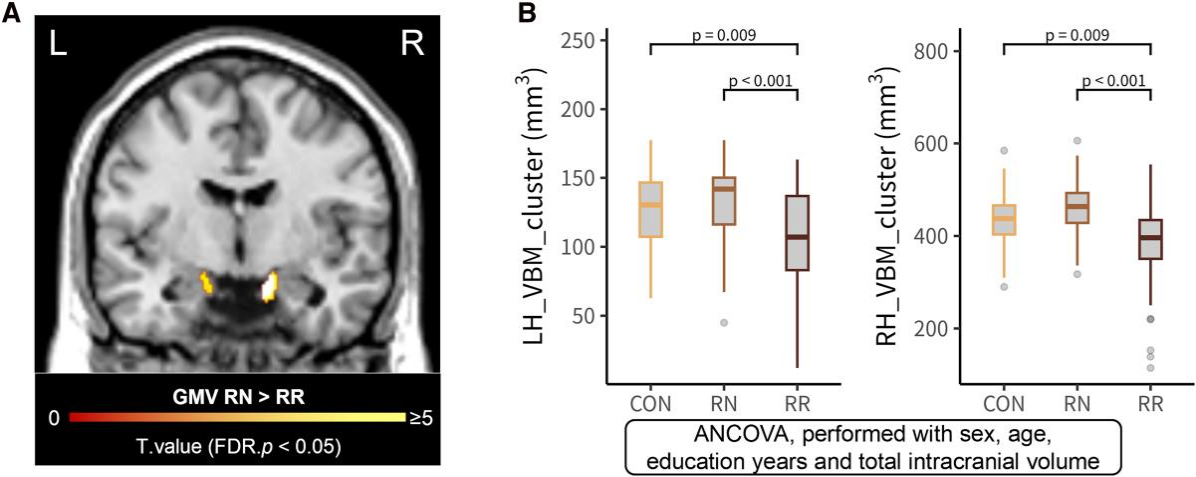
**Quantitative comparison of bilateral hippocampal atrophy across groups.** (**A**) VBM comparison showing greater bilateral hippocampal atrophy in RR patients relative to CON group (FDR-corrected *P* < 0.05). (**B**) Quantitative comparison of LH and RH volumes among the three groups. Statistical comparisons were performed using general linear models with FDR correction. Box plots display the median, IQR and whiskers extending to the most extreme data points within 1.5 × IQR. Group sample sizes: CON, *n* = 36; RN, *n* = 46; RR, *n* = 57.

**Figure 3:**
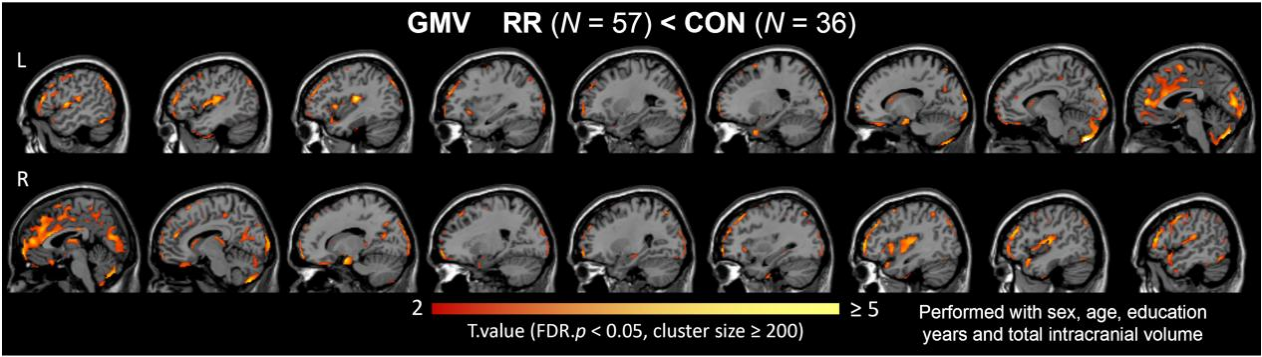
**Patterns of GM atrophy in the RR group compared with the CON group.** Statistical maps were thresholded at a cluster-level significance of *P* < 0.05, corrected for multiple comparisons using the FDR, with an underlying cluster-forming threshold of 200 contiguous voxels.

ROI analysis revealed significant between-group differences in the atrophy of the bilateral hippocampus after adjusting for age, sex and education level (Fig. 2; *P* < 0.001, FDR-corrected). *Post hoc* comparisons revealed a hierarchical pattern of GM atrophy: the RR group exhibited significantly greater atrophy in specific regions compared to both the CON and RN groups, while no significant differences were observed between the latter two. Moreover, the GM atrophy in the RR group was not limited to these regions; relative to the CON group, they demonstrated a more widespread pattern of volume loss, involving areas such as the insula, medial prefrontal cortex and ventral tegmental area (Fig. 3; *P* < 0.05, FDR-corrected). These findings indicate that in patients with rTLE and comorbid HS, significant atrophy is present not only in the ipsilateral hemisphere but also extensively across the brain. Notably, alongside the marked atrophy of the ipsilateral (right) hippocampus (RH), significant atrophy was also observed in the contralateral (left) hippocampus (LH). This pattern suggests the presence of cross-hemispheric network effects in unilateral TLE with HS.

### Detailed analysis of the hippocampal subregions

**Figure 4:**
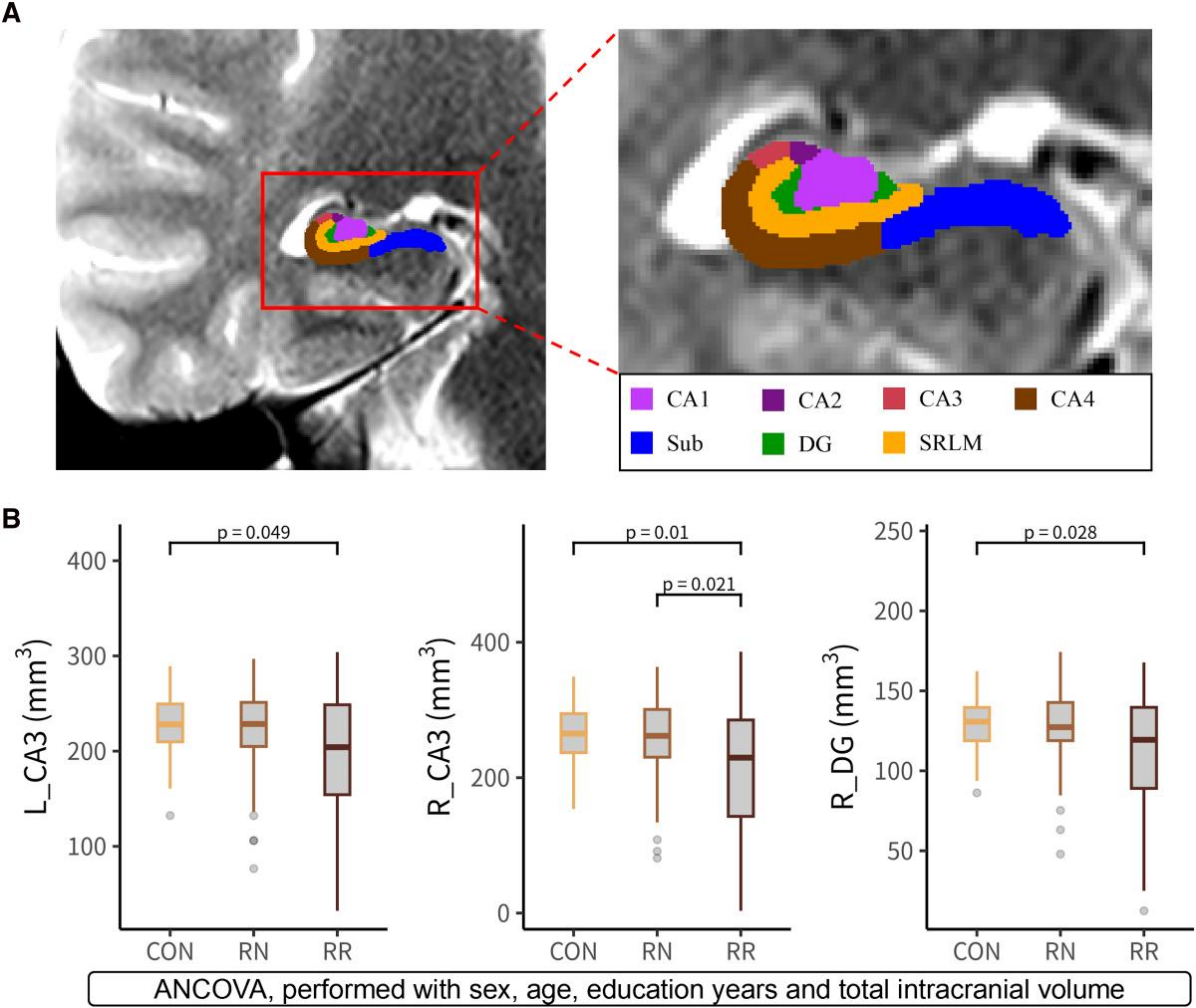
**Group differences in hippocampal subregions.** (**A**) Schematic representation of hippocampal subregions. (**B**) Quantitative comparisons of L_CA3, R_CA3 and R_DG volumes across groups. Statistical analyses were performed using general linear models with FDR correction. Box plots depict the median, IQR and whiskers extending to the most extreme data points within 1.5 × IQR. Sample sizes: CON, *n* = 36; RN, *n* = 46; RR, *n* = 57.

Volumetric comparisons of all hippocampal subregions, including CA1, CA2, CA3, the DG and the subiculum, are provided in Supplementary Fig. 1. After FDR correction, no significant differences were observed in CA1 or CA2 volumes among the groups. However, significant between-group differences emerged in other subregions (Fig. 4). Specifically, the RR group exhibited more pronounced atrophy in the left CA3 (L_CA3), right CA3 (R_CA3) and right DG (R_DG) compared to the CON group (all *P* < 0.05, FDR-corrected). In addition, R_CA3 atrophy was significantly greater in the RR group than in the RN group (*P* = 0.021, FDR-corrected). As core components of the classic hippocampal trisynaptic circuit, the CA3 and DG play essential roles in information encoding and pattern separation; thus, atrophy in these regions may directly contribute to cognitive impairments. Notably, the significant atrophy observed in the L_CA3 (contralateral) in the RR group suggests that unilateral HS can induce structural alterations in the opposite hemisphere, likely through cross-hemispheric network mechanisms.

### Cognitive performance

**Figure 5:**
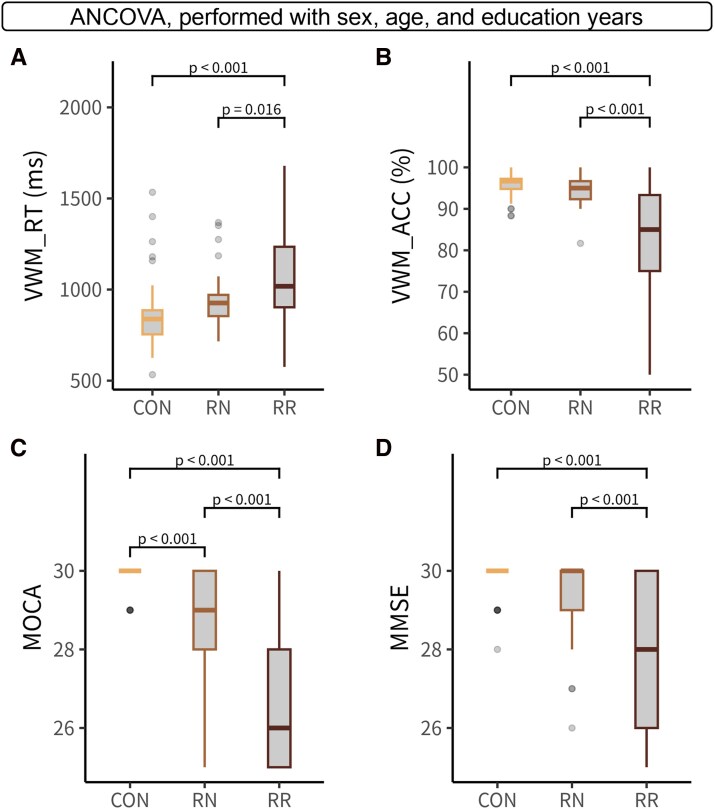
**Between-group comparisons of cognitive performance, adjusted for age, sex and education.** (**A**) VWM_RT. (**B**) VWM_ACC. (**C**) MoCA score. (**D**) MMSE score. Statistical comparisons were performed using general linear models with FDR correction. Box plots display the median, IQR and whiskers extending to the most extreme data points within 1.5 × IQR. Group sample sizes: CON, *n* = 36; RN, *n* = 46; RR, *n* = 57.

As shown in Fig. 5, after adjusting for age, sex and education level, we found significant between-group differences in VWM_RT, VWM_ACC, MoCA and MMSE (all *P* < 0.001, FDR-corrected). Relative to the RN group, RR patients demonstrated significantly longer VWM_RT (*P* = 0.016) and lower scores on the other three measures (VWM_ACC, MoCA and MMSE: all *P* < 0.001). In contrast to the CON group, RR patients showed significant reductions in all scores (all *P* < 0.001), while RN patients exhibited significantly lower MoCA scores (*P*  *<* 0.001). These cognitive results indicate that the presence of HS is associated with more severe VWM impairment and general cognitive decline. The dual impairment in both reaction time and accuracy observed in the RR group suggests deficits in both processing speed and precision of information maintenance.

### Associations between hippocampal subregion volumes and cognitive performance

**Figure 6:**
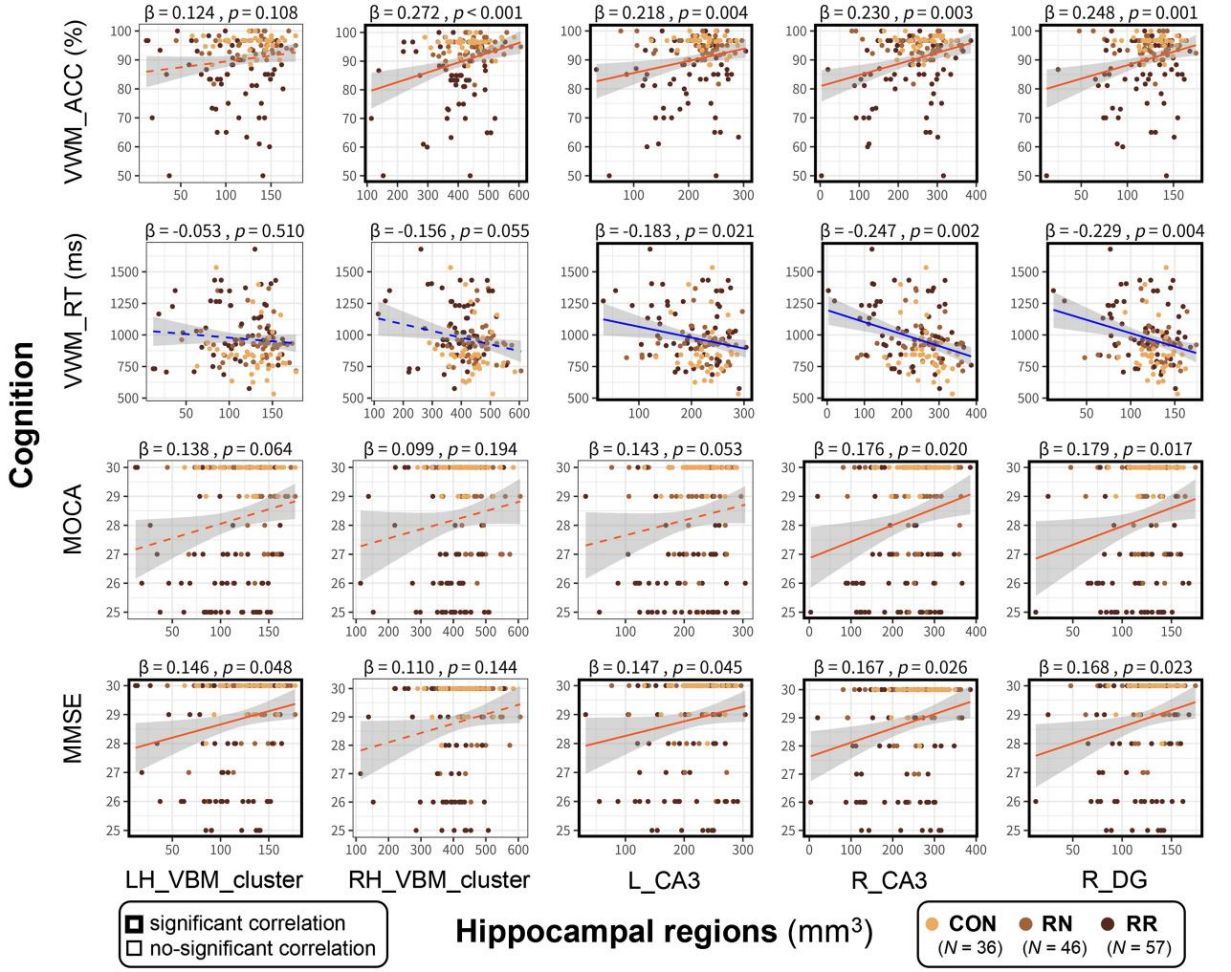
**Associations between hippocampal subregion volumes, VBM clusters and cognitive performance.** Scatter plots displaying the relationships between regional brain volumes and cognitive measures. Each point represents the cognitive index and GMV of each participant's corresponding brain region. The colour of the point represents different groups, while the colour and type of the line represent the direction and significance of partial correlation (red: positive correlation; blue: negative correlation; solid line: significant; dashed line: non-significant). Standardized regression coefficients (β) were derived from linear regression models adjusted for age, sex, education and TIV. Dashed lines represent the regression fit, and shaded areas indicate 95% confidence intervals.

Partial correlation analyses were conducted across the entire rTLE cohort (*n* = 103), adjusting for age, sex, education and TIV (Fig. 6). In addition, exploratory subgroup analyses were performed within the RR (*n* = 57) and RN (*n* = 46) groups separately; these results are provided in Supplementary Table 1, and the whole cohort analyses are presented in Supplementary Table 2. Positive correlations were observed between volumes of the RH subregions and cognitive scores: both R_CA3 and R_DG volumes correlated positively with MoCA, MMSE and VWM_ACC scores. Conversely, VWM_RT showed negative correlations with the volumes of these same subregions. In the left hemisphere, L_CA3 volume was positively correlated with VWM_ACC but negatively correlated with VWM_RT. Additionally, VWM_ACC was positively correlated with RH volume. These correlations further underscore the importance of hippocampal subregional structural integrity for cognitive function. The association between L_CA3 volume and VWM performance is particularly noteworthy, indicating that even the structural integrity of contralateral hippocampal subregions may influence cognitive outcomes in unilateral epilepsy. This highlights the need to consider the integrity of bilateral hippocampal networks when assessing epilepsy-related cognitive impairment.

## Discussion

This study integrated high-resolution MRI with systematic neuropsychological assessment to investigate the exacerbating effect of HS on VWM impairment and its underlying structural brain bases in patients with rTLE. The main findings can be summarized as follows: First, with age, sex, years of education and TIV included as covariates in the model, the RR group exhibited widespread cerebral GM atrophy. This involved regions such as the insula, medial prefrontal cortex and ventral tegmental area. The most severe atrophy was identified in the bilateral hippocampus and parahippocampal gyrus. Second, precise analysis of hippocampal subregions revealed specific atrophy in the L_CA3, R_CA3 and R_DG in the RR group compared to the CON group, with the atrophy in the R_CA3 being significantly greater than that in the RN group. Notably, a key finding was the significant atrophy observed in the contralateral hippocampus of the RR group, with specific atrophy noted in the L_CA3 subregion. Third, compared to the RN and CON groups, the RR group exhibited significantly worse VWM performance (characterized by prolonged reaction times and reduced accuracy) and basic cognitive function (lower MoCA and MMSE scores). Finally, correlation analyses indicated that the volume of specific hippocampal subregions has a significant relationship with cognitive performance. Consistent with the structural finding, the volume of L_CA3 showed a significant positive correlation with VWM_ACC and a negative correlation with VWM_RT. Similarly, the volumes of the R_CA3 and R_DG were significantly positively correlated with VWM_ACC and basic cognitive scores, and negatively correlated with VWM_RT. Collectively, these results indicate that in rTLE, HS is associated not only with more severe focal ipsilateral mesiotemporal structural damage (R_CA3 and DG) but also with contralateral hippocampal involvement, specifically of the L_CA3. This specific pattern of structural alterations, including bilateral CA3 pathology, likely constitutes the core pathological basis for the more pronounced VWM and cognitive dysfunction observed in these patients.

The findings of this study hold significant theoretical implications. First, we further support and extend the modern view of ‘epilepsy as a network disorder’.^27,28^ Traditionally, TLE-HS has been considered a focal disease primarily confined to the hippocampus.^29,30^ However, our whole-brain GMV analysis revealed widespread regional atrophy in the RR group extending beyond the temporal lobe. Crucially, the identification of atrophy in the contralateral L_CA3 subregion provides direct structural evidence for transhemispheric network effects in unilateral TLE-HS. This suggests that abnormal electrical activity originating from the sclerotic RH may exert pathological effects not only on ipsilateral and distal cortical nodes but also on the homologous structure in the contralateral hemisphere. The specific vulnerability of the contralateral CA3 subregion invites mechanistic speculation. Potential explanations include (i) trans-synaptic degeneration via established commissural pathways (e.g. the hippocampal commissure or ventral hippocampal commissure),^31^ where chronic hyperexcitability or excitotoxic insult from the sclerotic focus propagates to and damages connected neurons in the contralateral CA3; (ii) shared genetic, developmental or environmental vulnerabilities that predispose bilateral hippocampus, particularly the CA3 subregions, to independent or co-progressive pathology^32^; and (iii) secondary effects of widespread bilateral network dysfunction, where altered interhemispheric synchronization or disrupted default mode network integrity indirectly impacts the metabolic and structural health of the contralateral hippocampus.^33^ This evidence underscores a more profound and bilateral network disruption than previously often assumed for unilateral HS.^34^ Second, this study refines the anatomical specificity of the association between HS and cognitive dysfunction. Previous research has predominantly focused on the relationship between overall hippocampal volume, often ipsilateral and memory function.^35^ Our segmentation of hippocampal subregions indicates that atrophy in the CA3 subregions bilaterally is particularly closely associated with cognitive impairment. The CA3 and DG subregions occupy a critical position within the classic hippocampal trisynaptic circuit (mEC–DG–CA3–CA1).^36^ The DG serves as the primary gateway for cortical information into the hippocampus, performing pattern separation, the process of transforming similar inputs into distinct neural representations.^37,38^ CA3, with its extensive recurrent collateral network, is crucial for the rapid encoding of these separated patterns^39,40^ and for pattern completion, the ability to retrieve a complete memory from a partial cue.^41,42^ The preferential atrophy observed in the DG and CA3, rather than in the downstream CA1 subregion, which is more associated with output processing, suggests a disruption at the initial stages of information processing within the hippocampal circuit. This ‘input-level’ pathology in DG and CA3 could compromise the fidelity of information entering the memory system and its subsequent associative storage, providing a plausible neural substrate for the observed VWM deficits, which rely on the rapid and accurate maintenance of transient information. The significant correlations found between L_CA3 volume and VWM performance highlight that even contralateral hippocampal integrity, specifically of the CA3 subregion, is a relevant factor for cognitive function in unilateral TLE. This bilateral vulnerability of the CA3 region may be a key mechanism underlying the more generalized memory and cognitive deficits observed in TLE-HS patients.

In terms of clinical significance, this study underscores HS as an important biomarker for poor cognitive prognosis in TLE patients^43^ and reveals new aspects of its impact. First, the results support the systematic evaluation of hippocampal structure, extending beyond ipsilateral assessment to include bilateral subregion analysis (particularly CA3), alongside detailed cognitive function profiling, as a routine component in the clinical evaluation of TLE.^44,45^ The finding of contralateral CA3 involvement suggests that cognitive prognosis may be influenced by pathology extending beyond the epileptogenic hemisphere. This is crucial for comprehensive patient management and counselling. Second, identifying specific hippocampal subregions closely linked to cognitive impairment, now including the contralateral L_CA3, may inform future therapeutic strategies. The bilateral involvement of CA3 suggests that interventions aimed at modulating hippocampal network function or promoting resilience might need to consider both hemispheres. Furthermore, the finding that rTLE patients without HS (RN group) exhibited relatively milder cognitive deficits and no significant difference in brain atrophy patterns compared to controls suggests that accurately distinguishing the presence or absence of HS prior to considering surgical intervention for drug-resistant epilepsy has potential value in predicting postoperative cognitive risk. The discovery of contralateral atrophy adds a layer of complexity for pre-surgical planning and cognitive risk assessment.

However, this study also has several limitations that must be considered when interpreting the results and that point to directions for future research. First, the cross-sectional design cannot establish causality between HS (ipsilateral and contralateral), widespread brain atrophy and cognitive decline. The mechanism driving contralateral L_CA3 atrophy, whether it is a consequence of trans-synaptic degeneration, independent pathology, or a shared susceptibility, remains unclear and warrants longitudinal investigation. Second, the relatively small sample size of the CON group may have reduced statistical power. Future research should expand the healthy control sample to enhance the robustness of the results. Third, this study primarily focused on morphological changes in GMV. Future multimodal studies, combining functional and connectomic data, are needed to understand how contralateral hippocampal atrophy relates to interhemispheric functional connectivity and network dynamics in TLE-HS. Fourth, while the neuropsychological assessment covered basic cognition and VWM, a more comprehensive evaluation of material-specific memory (verbal/visual) could help clarify the functional implications of LH versus RH subregion atrophy.^46,47^

Based on the above findings and limitations, we propose the following directions for future research: (i) longitudinal studies are needed to track the progression of contralateral hippocampal changes and their temporal relationship with cognitive decline in TLE-HS; (ii) research should examine the molecular and cellular correlates of contralateral hippocampal pathology in surgical or post-mortem tissue to distinguish between different pathogenic mechanisms; and (iii) the role of the contralateral hippocampus, particularly the CA3 subregion, as a potential compensatory reserve or a co-determinant of cognitive outcomes should be explored in larger cohorts.

In conclusion, this study demonstrates that in patients with rTLE, comorbid HS is associated with more severe VWM deficits and extensive GM atrophy. A novel and key finding is the significant atrophy identified in the contralateral CA3 subregion and its correlation with VWM performance. These results advance our understanding of the neural substrates underlying cognitive comorbidity in TLE by revealing the bilateral, network-level structural pathology resulting from unilateral HS. This work underscores the central role of HS and its cross-hemispheric effects in the pathophysiology of epileptic networks and cognitive prognosis, providing a theoretical basis and identifying potential neuroanatomical targets (e.g. bilateral CA3) for future interventions aimed at mitigating cognitive dysfunction, as well as highlighting its implications for pre-surgical evaluation.

## Supplementary Material

fcag284_Supplementary_Data

## Data Availability

This study involved the re-analysis of existing clinical and imaging data. The original datasets are not publicly available due to patient confidentiality agreements under the approved ethics protocol. No custom code was generated for this study; all analyses were performed using standard, publicly available software packages (SPM12, HippUnfold v1.5.2). Further information about the data and conditions for access can be obtained by contacting the corresponding author.
