# Supplementary material for: Bilateral cornu ammonis 3–dominant mechanism of visual working memory impairment in right temporal lobe epilepsy and hippocampal sclerosis
Source: Brain Commun. 2026 Jul 20;8(4):fcag284. doi: 10.1093/braincomms/fcag284 (PMC13426314; doi:10.1093/braincomms/fcag284)
Supplement: fcag284_Supplementary_Data [file fcag284_supplementary_data.docx]

**Supplementary Table 1. Association of cognitive performance with cortical and hippocampal subfield volumes in RR, RN, and CON groups**

| **Group** | **Cognition** | **Brain region** | **std.β** | **nocor.*p*.value** | **FDR.*p*.value** |
| --- | --- | --- | --- | --- | --- |
| RR | VWM_ACC | LH_VBM_cluster | -0.093 | 0.5 | 0.877 |
| RN | VWM_ACC | LH_VBM_cluster | 0.23 | 0.177 | 0.877 |
| CON | VWM_ACC | LH_VBM_cluster | 0.101 | 0.42 | 0.877 |
| RR | VWM_ACC | RH_VBM_cluster | 0.083 | 0.535 | 0.906 |
| RN | VWM_ACC | RH_VBM_cluster | 0.172 | 0.347 | 0.877 |
| CON | VWM_ACC | RH_VBM_cluster | 0.136 | 0.346 | 0.877 |
| RR | VWM_ACC | L_CA1 | 0.139 | 0.282 | 0.877 |
| RN | VWM_ACC | L_CA1 | -0.095 | 0.558 | 0.913 |
| CON | VWM_ACC | L_CA1 | -0.036 | 0.79 | 0.98 |
| RR | VWM_ACC | L_CA2 | -0.014 | 0.919 | 0.983 |
| RN | VWM_ACC | L_CA2 | 0.012 | 0.944 | 0.983 |
| CON | VWM_ACC | L_CA2 | -0.17 | 0.172 | 0.877 |
| RR | VWM_ACC | L_CA3 | 0.166 | 0.204 | 0.877 |
| RN | VWM_ACC | L_CA3 | -0.15 | 0.382 | 0.877 |
| CON | VWM_ACC | L_CA3 | -0.136 | 0.277 | 0.877 |
| RR | VWM_ACC | L_CA4 | -0.152 | 0.233 | 0.877 |
| RN | VWM_ACC | L_CA4 | -0.109 | 0.495 | 0.877 |
| CON | VWM_ACC | L_CA4 | 0.222 | 0.07 | 0.877 |
| RR | VWM_ACC | L_DG | 0.201 | 0.142 | 0.877 |
| RN | VWM_ACC | L_DG | -0.183 | 0.267 | 0.877 |
| CON | VWM_ACC | L_DG | 0.107 | 0.395 | 0.877 |
| RR | VWM_ACC | R_CA1 | 0.189 | 0.156 | 0.877 |
| RN | VWM_ACC | R_CA1 | 0.081 | 0.669 | 0.966 |
| CON | VWM_ACC | R_CA1 | -0.125 | 0.304 | 0.877 |
| RR | VWM_ACC | R_CA2 | -0.047 | 0.731 | 0.968 |
| RN | VWM_ACC | R_CA2 | 0.01 | 0.955 | 0.983 |
| CON | VWM_ACC | R_CA2 | -0.141 | 0.242 | 0.877 |
| RR | VWM_ACC | R_CA3 | 0.148 | 0.265 | 0.877 |
| RN | VWM_ACC | R_CA3 | -0.072 | 0.674 | 0.966 |
| CON | VWM_ACC | R_CA3 | -0.082 | 0.5 | 0.877 |
| RR | VWM_ACC | R_CA4 | -0.032 | 0.818 | 0.983 |
| RN | VWM_ACC | R_CA4 | 0.025 | 0.889 | 0.983 |
| CON | VWM_ACC | R_CA4 | -0.042 | 0.742 | 0.969 |
| RR | VWM_ACC | R_DG | 0.198 | 0.134 | 0.877 |
| RN | VWM_ACC | R_DG | -0.164 | 0.344 | 0.877 |
| CON | VWM_ACC | R_DG | -0.11 | 0.385 | 0.877 |
| RR | VWM_RT | LH_VBM_cluster | 0.169 | 0.241 | 0.877 |
| RN | VWM_RT | LH_VBM_cluster | -0.023 | 0.881 | 0.983 |
| CON | VWM_RT | LH_VBM_cluster | -0.199 | 0.145 | 0.877 |
| RR | VWM_RT | RH_VBM_cluster | -0.049 | 0.725 | 0.968 |
| RN | VWM_RT | RH_VBM_cluster | -0.032 | 0.846 | 0.983 |
| CON | VWM_RT | RH_VBM_cluster | -0.141 | 0.373 | 0.877 |
| RR | VWM_RT | L_CA1 | -0.25 | 0.062 | 0.877 |
| RN | VWM_RT | L_CA1 | 0.001 | 0.994 | 0.994 |
| CON | VWM_RT | L_CA1 | -0.113 | 0.446 | 0.877 |
| RR | VWM_RT | L_CA2 | -0.02 | 0.892 | 0.983 |
| RN | VWM_RT | L_CA2 | -0.075 | 0.64 | 0.966 |
| CON | VWM_RT | L_CA2 | -0.019 | 0.889 | 0.983 |
| RR | VWM_RT | L_CA3 | -0.205 | 0.133 | 0.877 |
| RN | VWM_RT | L_CA3 | 0.156 | 0.309 | 0.877 |
| CON | VWM_RT | L_CA3 | -0.126 | 0.362 | 0.877 |
| RR | VWM_RT | L_CA4 | 0.109 | 0.416 | 0.877 |
| RN | VWM_RT | L_CA4 | 0.058 | 0.688 | 0.966 |
| CON | VWM_RT | L_CA4 | -0.174 | 0.204 | 0.877 |
| RR | VWM_RT | L_DG | -0.062 | 0.67 | 0.966 |
| RN | VWM_RT | L_DG | 0.056 | 0.704 | 0.966 |
| CON | VWM_RT | L_DG | -0.115 | 0.408 | 0.877 |
| RR | VWM_RT | R_CA1 | -0.228 | 0.103 | 0.877 |
| RN | VWM_RT | R_CA1 | -0.007 | 0.968 | 0.983 |
| CON | VWM_RT | R_CA1 | -0.046 | 0.733 | 0.968 |
| RR | VWM_RT | R_CA2 | -0.043 | 0.763 | 0.969 |
| RN | VWM_RT | R_CA2 | -0.109 | 0.476 | 0.877 |
| CON | VWM_RT | R_CA2 | 0.014 | 0.919 | 0.983 |
| RR | VWM_RT | R_CA3 | -0.25 | 0.07 | 0.877 |
| RN | VWM_RT | R_CA3 | 0.054 | 0.726 | 0.968 |
| CON | VWM_RT | R_CA3 | -0.203 | 0.126 | 0.877 |
| RR | VWM_RT | R_CA4 | -0.055 | 0.703 | 0.966 |
| RN | VWM_RT | R_CA4 | -0.051 | 0.754 | 0.969 |
| CON | VWM_RT | R_CA4 | -0.082 | 0.557 | 0.913 |
| RR | VWM_RT | R_DG | -0.242 | 0.079 | 0.877 |
| RN | VWM_RT | R_DG | 0.16 | 0.303 | 0.877 |
| CON | VWM_RT | R_DG | -0.109 | 0.437 | 0.877 |
| RR | MOCA | LH_VBM_cluster | 0.006 | 0.962 | 0.983 |
| RN | MOCA | LH_VBM_cluster | -0.142 | 0.346 | 0.877 |
| CON | MOCA | LH_VBM_cluster | -0.004 | 0.976 | 0.983 |
| RR | MOCA | RH_VBM_cluster | -0.117 | 0.365 | 0.877 |
| RN | MOCA | RH_VBM_cluster | -0.331 | 0.036 | 0.877 |
| CON | MOCA | RH_VBM_cluster | -0.132 | 0.402 | 0.877 |
| RR | MOCA | L_CA1 | 0.14 | 0.265 | 0.877 |
| RN | MOCA | L_CA1 | -0.26 | 0.064 | 0.877 |
| CON | MOCA | L_CA1 | 0.164 | 0.263 | 0.877 |
| RR | MOCA | L_CA2 | 0.133 | 0.332 | 0.877 |
| RN | MOCA | L_CA2 | -0.064 | 0.68 | 0.966 |
| CON | MOCA | L_CA2 | 0.041 | 0.767 | 0.969 |
| RR | MOCA | L_CA3 | 0.025 | 0.844 | 0.983 |
| RN | MOCA | L_CA3 | -0.154 | 0.304 | 0.877 |
| CON | MOCA | L_CA3 | 0.096 | 0.485 | 0.877 |
| RR | MOCA | L_CA4 | 0.022 | 0.857 | 0.983 |
| RN | MOCA | L_CA4 | 0.028 | 0.843 | 0.983 |
| CON | MOCA | L_CA4 | 0.07 | 0.611 | 0.966 |
| RR | MOCA | L_DG | 0.125 | 0.35 | 0.877 |
| RN | MOCA | L_DG | -0.02 | 0.893 | 0.983 |
| CON | MOCA | L_DG | 0.264 | 0.049 | 0.877 |
| RR | MOCA | R_CA1 | 0.123 | 0.345 | 0.877 |
| RN | MOCA | R_CA1 | -0.27 | 0.098 | 0.877 |
| CON | MOCA | R_CA1 | 0.007 | 0.956 | 0.983 |
| RR | MOCA | R_CA2 | 0.119 | 0.365 | 0.877 |
| RN | MOCA | R_CA2 | -0.058 | 0.698 | 0.966 |
| CON | MOCA | R_CA2 | -0.012 | 0.927 | 0.983 |
| RR | MOCA | R_CA3 | 0.095 | 0.463 | 0.877 |
| RN | MOCA | R_CA3 | -0.178 | 0.231 | 0.877 |
| CON | MOCA | R_CA3 | 0.16 | 0.226 | 0.877 |
| RR | MOCA | R_CA4 | 0.142 | 0.291 | 0.877 |
| RN | MOCA | R_CA4 | -0.103 | 0.516 | 0.884 |
| CON | MOCA | R_CA4 | 0.058 | 0.677 | 0.966 |
| RR | MOCA | R_DG | 0.097 | 0.452 | 0.877 |
| RN | MOCA | R_DG | -0.256 | 0.089 | 0.877 |
| CON | MOCA | R_DG | 0.141 | 0.309 | 0.877 |
| RR | MMSE | LH_VBM_cluster | -0.034 | 0.81 | 0.983 |
| RN | MMSE | LH_VBM_cluster | 0.005 | 0.972 | 0.983 |
| CON | MMSE | LH_VBM_cluster | 0.206 | 0.135 | 0.877 |
| RR | MMSE | RH_VBM_cluster | -0.127 | 0.343 | 0.877 |
| RN | MMSE | RH_VBM_cluster | -0.126 | 0.377 | 0.877 |
| CON | MMSE | RH_VBM_cluster | 0.124 | 0.441 | 0.877 |
| RR | MMSE | L_CA1 | 0.128 | 0.326 | 0.877 |
| RN | MMSE | L_CA1 | -0.114 | 0.367 | 0.877 |
| CON | MMSE | L_CA1 | 0.012 | 0.937 | 0.983 |
| RR | MMSE | L_CA2 | 0.098 | 0.491 | 0.877 |
| RN | MMSE | L_CA2 | 0.06 | 0.666 | 0.966 |
| CON | MMSE | L_CA2 | -0.013 | 0.928 | 0.983 |
| RR | MMSE | L_CA3 | 0.052 | 0.698 | 0.966 |
| RN | MMSE | L_CA3 | -0.098 | 0.466 | 0.877 |
| CON | MMSE | L_CA3 | 0.093 | 0.505 | 0.877 |
| RR | MMSE | L_CA4 | -0.093 | 0.471 | 0.877 |
| RN | MMSE | L_CA4 | -0.099 | 0.427 | 0.877 |
| CON | MMSE | L_CA4 | -0.023 | 0.872 | 0.983 |
| RR | MMSE | L_DG | 0.112 | 0.419 | 0.877 |
| RN | MMSE | L_DG | 0.019 | 0.881 | 0.983 |
| CON | MMSE | L_DG | 0.134 | 0.338 | 0.877 |
| RR | MMSE | R_CA1 | 0.111 | 0.413 | 0.877 |
| RN | MMSE | R_CA1 | -0.205 | 0.16 | 0.877 |
| CON | MMSE | R_CA1 | 0.039 | 0.775 | 0.971 |
| RR | MMSE | R_CA2 | 0.043 | 0.752 | 0.969 |
| RN | MMSE | R_CA2 | 0.068 | 0.608 | 0.966 |
| CON | MMSE | R_CA2 | -0.081 | 0.55 | 0.913 |
| RR | MMSE | R_CA3 | 0.053 | 0.692 | 0.966 |
| RN | MMSE | R_CA3 | -0.058 | 0.66 | 0.966 |
| CON | MMSE | R_CA3 | 0.138 | 0.307 | 0.877 |
| RR | MMSE | R_CA4 | 0.095 | 0.497 | 0.877 |
| RN | MMSE | R_CA4 | 0.033 | 0.817 | 0.983 |
| CON | MMSE | R_CA4 | -0.005 | 0.97 | 0.983 |
| RR | MMSE | R_DG | 0.067 | 0.618 | 0.966 |
| RN | MMSE | R_DG | -0.151 | 0.265 | 0.877 |
| CON | MMSE | R_DG | 0.117 | 0.41 | 0.877 |

Abbreviations: CON, healthy controls; RN, patients with right temporal lobe epilepsy without right hippocampal sclerosis; RR, patients with right temporal lobe epilepsy with right hippocampal sclerosis; VWM_ACC, visual working memory accuracy; VWM_RT, visual working memory reaction time; MOCA, Montreal Cognitive Assessment; MMSE, Mini-Mental State Examination; LH_VBM_cluster, left hippocampal voxel-based morphometry analysis cluster; RH_VBM_cluster, right hippocampal voxel-based morphometry analysis cluster; L_CA1–4, left cornu ammonis subfields 1–4; R_CA1–4, right cornu ammonis subfields 1–4; L_DG, left dentate gyrus; R_DG, right dentate gyrus; std.β, standardized β coefficient; nocor.*p*.value, uncorrected *p*-value; FDR.*p*.value, false discovery rate-adjusted *p*-value.

**Supplementary Table 2. Associations Between Cognitive Performance and Hippocampal Subfield Volumes in All Participants**

| **Cognition** | **Brain region** | **std.β** | **nocor.*p*.value** | **FDR.*p*.value** |
| --- | --- | --- | --- | --- |
| VWM_ACC | LH_VBM_cluster | 0.124 | 0.108 | 0.173 |
| VWM_ACC | RH_VBM_cluster | 0.272 | 0 | 0.022 |
| VWM_ACC | L_CA1 | 0.175 | 0.023 | 0.079 |
| VWM_ACC | L_CA2 | 0.074 | 0.357 | 0.46 |
| VWM_ACC | L_CA3 | 0.218 | 0.004 | 0.025 |
| VWM_ACC | L_CA4 | -0.066 | 0.395 | 0.486 |
| VWM_ACC | L_DG | 0.056 | 0.475 | 0.543 |
| VWM_ACC | R_CA1 | 0.214 | 0.007 | 0.038 |
| VWM_ACC | R_CA2 | 0.02 | 0.798 | 0.798 |
| VWM_ACC | R_CA3 | 0.23 | 0.003 | 0.025 |
| VWM_ACC | R_CA4 | 0.044 | 0.584 | 0.609 |
| VWM_ACC | R_DG | 0.248 | 0.001 | 0.025 |
| VWM_RT | LH_VBM_cluster | -0.053 | 0.51 | 0.544 |
| VWM_RT | RH_VBM_cluster | -0.156 | 0.055 | 0.119 |
| VWM_RT | L_CA1 | -0.235 | 0.003 | 0.025 |
| VWM_RT | L_CA2 | -0.103 | 0.214 | 0.293 |
| VWM_RT | L_CA3 | -0.183 | 0.021 | 0.079 |
| VWM_RT | L_CA4 | 0.054 | 0.499 | 0.544 |
| VWM_RT | L_DG | -0.061 | 0.451 | 0.538 |
| VWM_RT | R_CA1 | -0.237 | 0.004 | 0.025 |
| VWM_RT | R_CA2 | -0.085 | 0.294 | 0.392 |
| VWM_RT | R_CA3 | -0.247 | 0.002 | 0.025 |
| VWM_RT | R_CA4 | -0.113 | 0.171 | 0.256 |
| VWM_RT | R_DG | -0.229 | 0.004 | 0.025 |
| MOCA | LH_VBM_cluster | 0.138 | 0.064 | 0.133 |
| MOCA | RH_VBM_cluster | 0.099 | 0.194 | 0.279 |
| MOCA | L_CA1 | 0.127 | 0.087 | 0.155 |
| MOCA | L_CA2 | 0.17 | 0.026 | 0.079 |
| MOCA | L_CA3 | 0.143 | 0.053 | 0.119 |
| MOCA | L_CA4 | 0.03 | 0.689 | 0.704 |
| MOCA | L_DG | 0.051 | 0.505 | 0.544 |
| MOCA | R_CA1 | 0.14 | 0.069 | 0.133 |
| MOCA | R_CA2 | 0.125 | 0.1 | 0.166 |
| MOCA | R_CA3 | 0.176 | 0.02 | 0.079 |
| MOCA | R_CA4 | 0.134 | 0.083 | 0.153 |
| MOCA | R_DG | 0.179 | 0.017 | 0.079 |
| MMSE | LH_VBM_cluster | 0.146 | 0.048 | 0.119 |
| MMSE | RH_VBM_cluster | 0.11 | 0.144 | 0.224 |
| MMSE | L_CA1 | 0.144 | 0.05 | 0.119 |
| MMSE | L_CA2 | 0.165 | 0.03 | 0.085 |
| MMSE | L_CA3 | 0.147 | 0.045 | 0.119 |
| MMSE | L_CA4 | -0.055 | 0.46 | 0.538 |
| MMSE | L_DG | 0.068 | 0.364 | 0.46 |
| MMSE | R_CA1 | 0.14 | 0.067 | 0.133 |
| MMSE | R_CA2 | 0.097 | 0.198 | 0.279 |
| MMSE | R_CA3 | 0.167 | 0.026 | 0.079 |
| MMSE | R_CA4 | 0.128 | 0.094 | 0.161 |
| MMSE | R_DG | 0.168 | 0.023 | 0.079 |

Abbreviations: VWM_ACC, visual working memory accuracy; VWM_RT, visual working memory reaction time; MOCA, Montreal Cognitive Assessment; MMSE, Mini-Mental State Examination; LH_VBM_cluster, left hippocampal voxel-based morphometry analysis cluster; RH_VBM_cluster, right hippocampal voxel-based morphometry analysis cluster; L_CA1–4, left cornu ammonis subfields 1–4; R_CA1–4, right cornu ammonis subfields 1–4; L_DG, left dentate gyrus; R_DG, right dentate gyrus; std.β, standardized β coefficient; nocor.*p*.value, uncorrected *p*-value; FDR.p.value, false discovery rate-adjusted *p*-value.


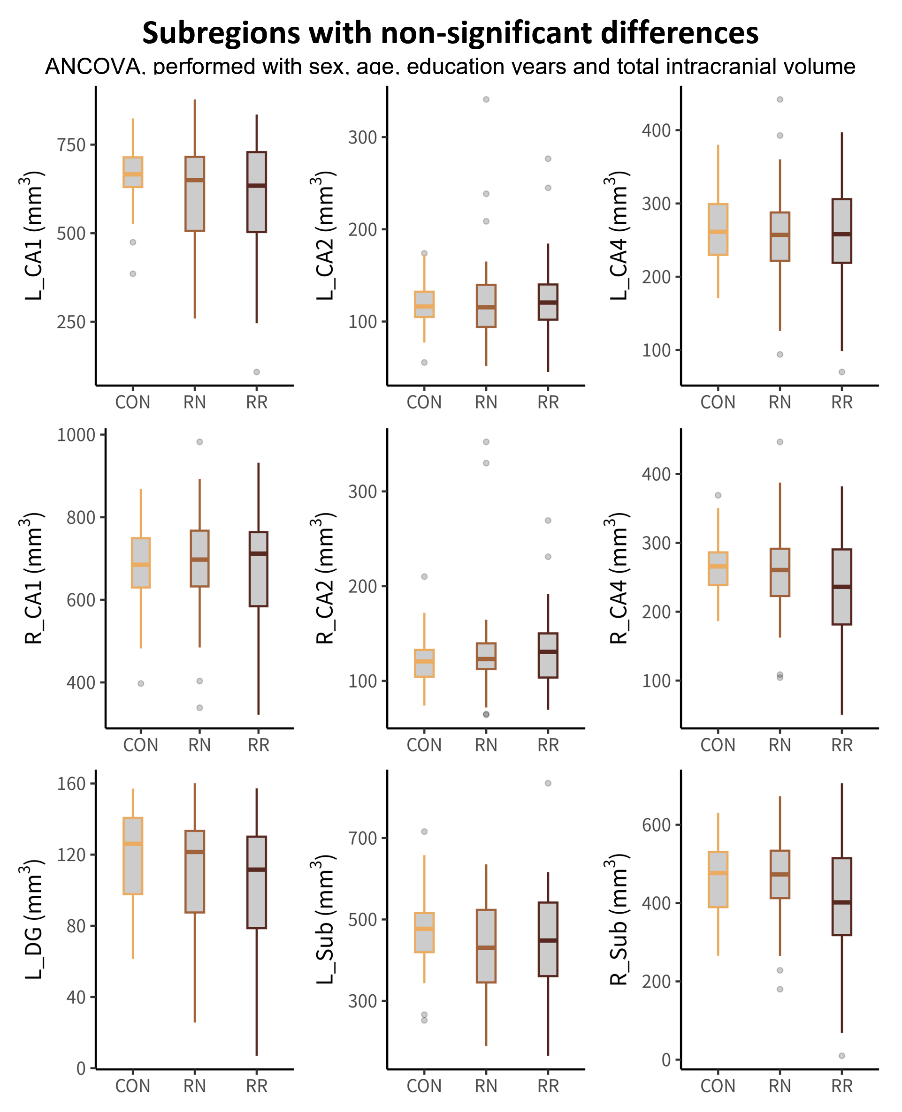


**Supplementary Figure 1. Group differences in non-significant hippocampal subregions.** Quantitative comparison of left and right CA1 (L_CA1, R_CA1), CA2 (L_CA2, R_CA2), CA4 (L_CA4, R_CA4), dentate gyrus (L_DG, R_DG), and subiculum (L_SUB, R_SUB) volumes across groups. Statistical comparisons were performed using ANCOVAwith false discovery rate (FDR) correction. Box plots display the median, interquartile range (IQR), and whiskers extending to the most extreme data points within 1.5 × IQR. Group sample sizes: CON, n = 36; RN, n = 46; RR, n = 57. Abbreviations: CON, healthy controls; RN, right temporal lobe epilepsy without hippocampal sclerosis; RR, right temporal lobe epilepsy with hippocampal sclerosis; L, left; R, right; CA, cornu ammonis; DG, dentate gyrus; SUB, subiculum.
